# Speckle Tracking Echocardiography: New Ways of Translational Approaches in Preeclampsia to Detect Cardiovascular Dysfunction

**DOI:** 10.3390/ijms21031162

**Published:** 2020-02-10

**Authors:** Kristin Kräker, Till Schütte, Jamie O’Driscoll, Anna Birukov, Olga Patey, Florian Herse, Dominik N. Müller, Basky Thilaganathan, Nadine Haase, Ralf Dechend

**Affiliations:** 1 Experimental and Clinical Research Center, a joint cooperation between the Max – Delbrück—Center for Molecular Medicine and the Charité—Universitätsmedizin Berlin, 13125 Berlin, Germany; kristin.kraeker@charite.de; 2 Max – Delbrück—Center for Molecular Medicine in the Helmholtz Association, 13125 Berlin, Germany; 3 Charité—Universitätsmedizin Berlin, corporate member of Freie Universität Berlin, Humboldt—Universität zu Berlin, and Berlin Institute of Health, 10117 Berlin, Germany; 4 Berlin Institute of Health (BIH), 10178 Berlin, Germany; 5 DZHK (German Centre for Cardiovascular Research), partner site Berlin, 10785 Berlin, Germany; 6 Institute of Pharmacology, Charité—Universitätsmedizin Berlin, corporate member of Freie Universität Berlin, Humboldt—Universität zu Berlin, and Berlin Institute of Health, 10115 Berlin, Germany; 7 Molecular & Clinical Sciences Research Institute, St George’s University of London, London SW17 0RE, UK; 8 Fetal Medicine Unit, St. George’s University Hospitals NHS Foundation Trust, London SW17 0QT, UK; 9 Canterbury Christ Church University, School of Human and Life Sciences, Kent CT1 1QU, UK; 10 Department of Molecular Epidemiology, German Institute of Human Nutrition Potsdam-Rehbrücke, 14558 Nuthetal, Germany; 11 Brompton Centre for Fetal Cardiology, Royal Brompton and Harefield Hospitals NHS Foundation Trust, London SW3 6NP, UK; 12 HELIOS-Klinikum, 13125 Berlin, Germany

**Keywords:** preeclampsia, pregnancy, speckle tracking echocardiography, cardiovascular dysfunction, animal models of human disease

## Abstract

Several studies have shown that women with a preeclamptic pregnancy exhibit an increased risk of cardiovascular disease. However, the underlying molecular mechanisms are unknown. Animal models are essential to investigate the causes of this increased risk and have the ability to assess possible preventive and therapeutic interventions. Using the latest technologies such as speckle tracking echocardiography (STE), it is feasible to map subclinical changes in cardiac diastolic and systolic function as well as structural changes of the maternal heart. The aim of this work is to compare cardiovascular changes in an established transgenic rat model with preeclampsia-like pregnancies with findings from human preeclamptic pregnancies by STE. The same algorithms were used to evaluate and compare the changes in echoes of human and rodents. Parameters of functionality such as global longitudinal strain (animal −23.54 ± 1.82% vs. −13.79 ± 0.57%, human −20.60 ± 0.47% vs. −15.45 ± 1.55%) as well as indications of morphological changes such as relative wall thickness (animal 0.20 ± 0.01 vs. 0.25 ± 0.01, human 0.34 ± 0.01 vs. 0.40 ± 0.02) are significantly altered in both species after preeclamptic pregnancies. Thus, the described rat model simulates the human situation quite well and is a valuable tool for future investigations regarding cardiovascular changes. STE is a unique technique that can be applied in animal models and humans with a high potential to uncover cardiovascular maladaptation and subtle pathologies.

## 1. Introduction

A pathological pregnancy, including preterm birth, gestational diabetes mellitus, and preeclampsia (PE), is the first gender-specific risk factor for cardiovascular disease (CVD) later in life [1]. PE, with its typical symptoms including the onset of hypertension and signs of end-organ damage, has a fourfold increase regarding the risk for long-term CVD [2]. In the last years, cardiovascular changes during a preeclamptic pregnancy and especially persistent alterations in maternal cardiac structure and function moved into the focus of scientific research [3]. It has been demonstrated that former preeclamptic women show relaxation abnormalities, diastolic dysfunction, left ventricular (LV) hypertrophy [4,5,6], and abnormal response to volume expansion and exercise [7,8,9]. In most of the studies, symptoms are rather mild and sometimes even asymptomatic. It remains unclear whether the increased risk for future CVD is related to PE or predisposing and preexisting factors [10], and thus PE represents a window to compromised future cardiovascular health. The mechanism for pathological functional and structural remodeling in the maternal heart after a pathological pregnancy is also not fully understood. 

For this reason, representative animal models are of increasing importance. A well-established rodent model for PE is the rat model, whose female is transgenic for human angiotensinogen and is mated with a transgenic male for human renin. Both animals are phenotypically inconspicuous until mating. Interestingly, dams develop typical symptoms of PE such as high blood pressure and albuminuria in the last trimester of pregnancy [11,12]. In addition, placenta-induced pathology with intrauterine growth retardation [12,13], elevated sFlt-1 levels [14], and existing AT1 autoantibodies [15] are presented in this animal model. Although PE is not caused by a monocausal pathology, this model reflects appropriately the human aspects of the disease. The use of rodent models such as this will help us to understand the mechanisms of disease-related alterations in cardiac function and structure that influence maternal long-term health after a pathological pregnancy. Furthermore, potential novel preventive and therapeutic strategies can be evaluated. Nevertheless, the gap between animal models and the human disease is wide, and translational research strategies are warranted to narrow the bridge.

In this study, we tested the hypothesis of whether the transgenic rat model is suitable for the investigation of the cardiovascular burden after PE. Recently, we showed that the preeclamptic rat suffers from hypertrophy and diastolic dysfunction [16]. Next, we compared this animal model with the systolic and the diastolic functions of the human maternal heart after a preeclamptic pregnancy. Speckle tracking echocardiography (STE), a state-of-the-art technique considered as the gold standard for early detection of cardiac dysfunction, is the current key tool for the non-invasive measurement of changes in cardiac function and structure. In this ultrasound imaging technique, the movement of heart tissue is analyzed using the naturally occurring speckle pattern in the heart muscle or in the blood. This mixture of interference patterns and natural acoustic reflections makes it possible to document the movement of the heart muscle by defining vectors and speed. These reflections are also known as speckles, which have a unique pattern for each region of the myocardium and make it possible to follow the region from one image to the next. Strain is defined as the percentage change in the dimension of an object compared to the original shape. Similarly, strain rate can be defined as the speed at which the deformation occurs. When applied to the LV, the deformation is defined by the three strains: longitudinal, circumferential, and radial. There is a systolic longitudinal and circumferential shortening and a radial thickening. Therefore, longitudinal and circumferential parameters have negative values, whereas radial parameters have positive values.

Formerly preeclamptic women were examined by STE 50 weeks after delivery and compared with age-matched controls. Transgenic rats were subjected to echocardiography and the same post processing algorithms four weeks after a preeclamptic pregnancy, which corresponds to two years in the human situation [17].

## 2. Results

**Figure 1:**
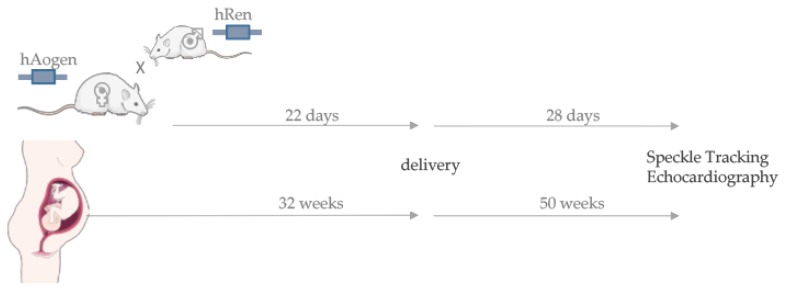
Formerly preeclamptic women and rats from a transgenic animal model were characterized postpartum regarding cardiac alterations in function and structure. Early onset preeclamptic women showed a lower gestational age than controls but were matched on scanning time after delivery.

Speckle tracking echocardiography (STE) was performed four weeks postpartum in formerly pregnant rats with PE-specific symptoms such as high blood pressure and albuminuria. In comparison, the same method was performed in formerly preeclamptic women 50 weeks after delivery (Figure 1). Both former preeclamptic species (PE) were compared with matched controls after healthy pregnancy (control). 

### 2.1. Speckle Tracking Echocardiography in the Transgenic Animal Model Simulates the Human Situation

**Figure 2:**
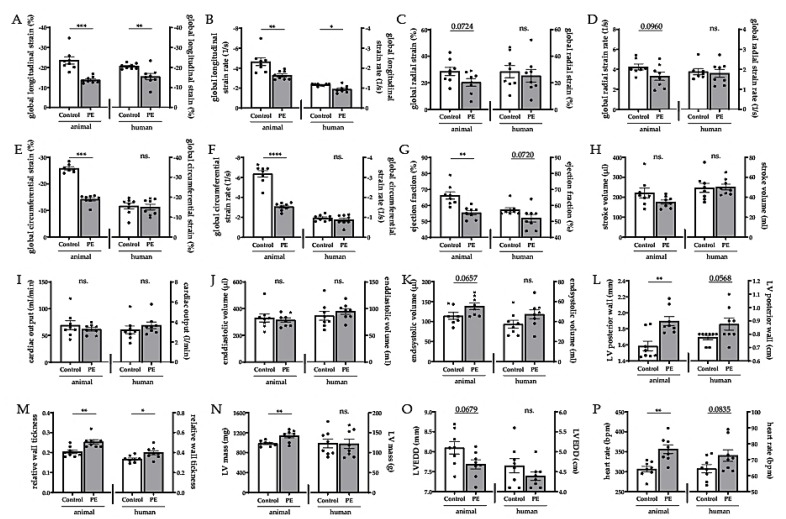
The transgenic rat model simulates cardiac alterations of a human preeclamptic pregnancy. Global longitudinal strain (**A**) and global longitudinal strain rate (**B**) were decreased after preeclampsia (PE). Global radial strain (**C**) and the corresponding strain rate (**D**) were not altered after PE. Global circumferential strain (**E**) and global circumferential strain rate (**F**) were reduced in the post-PE animals but not in the human cohort. Ejection fraction was reduced in animals and showed the same trend in the human PE data (**G**). Stroke volume (**H**), cardiac output (**I**), end-diastolic (**J**), and end-systolic volume (**K**) were not altered in either species after PE. Left ventricle (LV) posterior wall (**L**) and relative wall thickness (**M**) were increased due to PE in both species. LV mass (**N**) was only increased in the post-PE animals. LV end-diastolic diameter (**O**) was unaltered. Heart rate was higher in PE animals and showed increasing trends in humans (**P**). Mean values ± SEM, unpaired students t-test, ns. Non-significant, * *p* < 0.05, ** *p* < 0.01, *** *p* < 0.001, **** *p* < 0.0001.

The most important readout in STE is the global strain data. It describes the degree of deformation of the myocardium in different directions. The most sensitive parameter, the global longitudinal strain (GLS), was reduced after PE in both the animal model (control −23.5 ± 1.8% vs. PE −13.8 ± 0.6%) and the human (control −20.6 ± 0.5% vs. PE −15.5 ± 1.6%) postpartum situation (Figure 2A), and it was the same regarding the global longitudinal strain rate (Figure 2B). The global radial strain (Figure 2C) and the corresponding strain rate (Figure 2D) showed only a decreased tendency in the animal post-PE model and were unchanged in the human situation. The global circumferential strain (Figure 2E) and the corresponding strain rate (Figure 2F) were reduced in the animal model but showed no changes after human preeclamptic pregnancy. Moreover, former PE animals demonstrated a clear reduction of the ejection fraction (EF) with control 66.3 ± 2.3% vs. PE 55.5 ± 1.3%. The human data confirm the trend (Figure 2G). The stroke volume (Figure 2H) as well as the cardiac output (Figure 2I) and the end diastolic volume (Figure 2J) were not altered postpartum in any of the species. The end systolic volume only showed a slight increase in the animal model but not in the human postpartum situation (Figure 2K). In addition, echocardiography provided the first initial evidence of morphological changes. The posterior wall was clearly thickened in the animal post-PE model; in the human situation, it showed a borderline *p*-value of 0.06 (Figure 2L). If the relative wall thickness was considered, both species showed a postpartum increase (Figure 2M) with control 0.20 ± 0.01 vs. PE 0.25 ± 0.01 in the animal model and control 0.34 ± 0.01 vs. PE 0.40 ± 0.02 in the human postpartum situation. Interestingly, formerly preeclamptic animals showed an increase in LV masses (control 983.5 ± 19.0 mg vs. PE 1138.0 ± 40.6 mg); however, formerly preeclamptic women did not (Figure 2N). The LV diameter in the end diastole was not significantly changed in either species (Figure 2O). The heart rate was significantly increased in the animal model after preeclamptic pregnancy (control 306.6 ± 6.8 bpm vs. PE 356.2 ± 11.0 bpm). With a *p*-value of 0.08, this trend was also evident in the human situation (Figure 2P).

**Figure 3:**
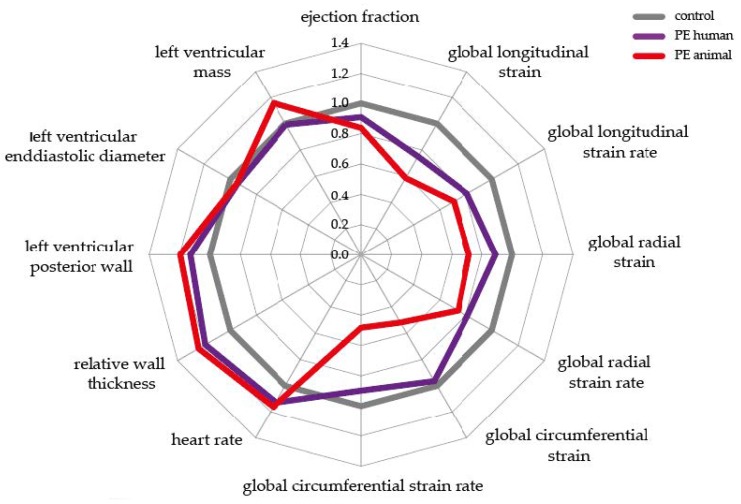
Relative values of post-preeclamptic changes in the animal model and in the human situation. Controls of each species were normalized to one. Grey line = controls, purple line = PE human, red line = PE animal; PE preeclampsia.

Figure 3 summarizes the described measurements in relative changes after a preeclamptic pregnancy compared to the healthy controls by a spider’s web plot. Here, the examined parameters are noted in the corners. The controls were normalized to the reference value 1.0, which is shown as a grey line. Changes in the animal post-PE model are reflected by the red line, and the purple line refers to the changes in the human post-PE situation compared to healthy controls. Concludingly, similar deviations after pathological pregnancy in both species can be observed. Several functional parameters, such as EF, were reduced, and parameters representing structural remodeling, such as relative wall thickness, were increased in both species compared to controls.

### 2.2. Intraobserver Variability Shows an Excellent Correlation within One Observer

**Figure 4:**
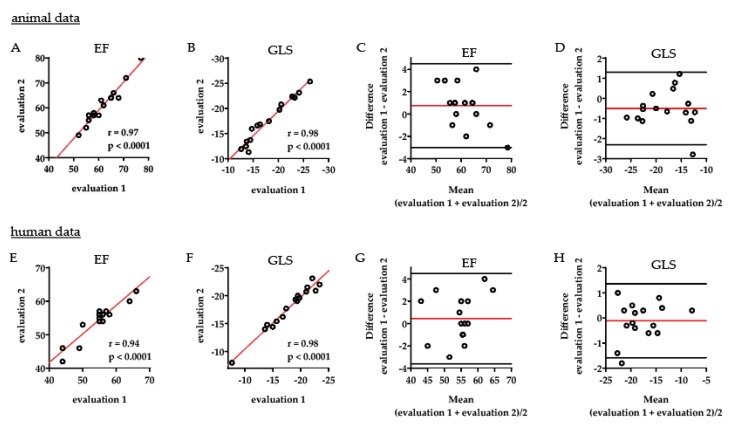
Intraobserver comparison. In analyses of animal data, there was an excellent correlation between the two repeated evaluations of ejection fraction, r = 0.97, *p* < 0.0001 (**A**), and global longitudinal strain, r = 0.98, *p* < 0.0001 (**B**) within one observer. The excellent agreement between the two evaluations was substantiated in the corresponding Bland–Altman plots, which showed only a marginal bias, mean difference (95% CI for limits of agreement) 0.75 (4.50 to −3.00) for ejection fraction (**C**) and −0.50 (1.30 to −2.31) for global longitudinal strain (**D**). In human data analyses, there was likewise an excellent intraobserver correlation regarding measurements of ejection fraction, r = 0.94, *p* < 0.0001 (**E**), and global longitudinal strain, r = 0.98, *p* < 0.0001 (**F**). Only minor bias was seen in the corresponding Bland–Altman plots: 0.44 (4.48 to −3.61) for repeated evaluation of human ejection fraction (**G**) and −0.11 (1.36 to −1.58) for global longitudinal strain (**H**). EF = ejection fraction, GLS = global longitudinal strain.

Analyses of the intraobserver variability in the animal data showed an excellent correlation between two repeated evaluations of EF, r = 0.97, *p* < 0.0001 (Figure 4A), and GLS, r = 0.98, *p* < 0.0001 (Figure 4B) within one observer. The very strong agreement between repeated evaluations was substantiated in the corresponding Bland–Altman plots, which showed only a marginal bias mean difference (95% CI for limits of agreement): 0.75 (4.50 to −3.00) for EF (Figure 4C) and −0.50 (1.30 to −2.31) for GLS (Figure 4D). In the human data analyses, there was likewise an excellent intraobserver correlation regarding measurements of EF, r = 0.94, *p* < 0.0001 (Figure 4E), and GLS, r = 0.98, *p* < 0.0001 (Figure 4F). Only a minor bias was seen in the corresponding Bland–Altman plots: 0.44 (4.48 to −3.61) for repeated evaluation of human EF (Figure 4G) and −0.11 (1.36 to −1.58) for human GLS (Figure 4H). The intraclass correlation coefficients (ICCs) for reliability of the evaluations were excellent both in animal and in human analyses (95% CI): 0.96 (0.89 to 0.99) for animal EF, 0.97 (0.92 to 0.99) for animal GLS, 0.93 (0.83 to 0.98) for human EF, 0.98 (0.95 to 0.99) for human GLS evaluations.

### 2.3. Interobserver Comparison Displays Strong Correlation between Two Different Observers

**Figure 5:**
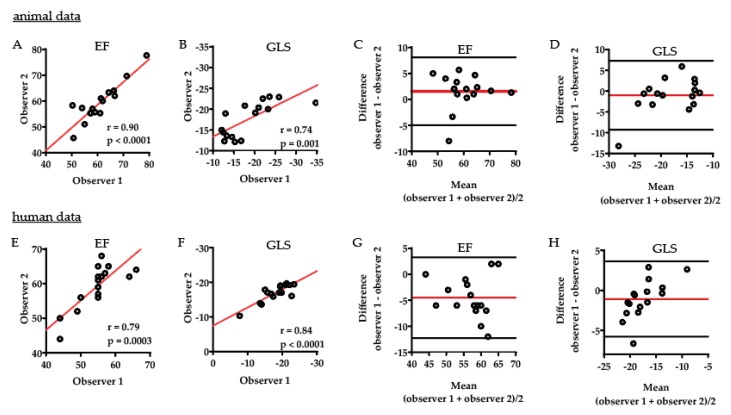
Interobserver comparison. In analysis by two experts, variability comparison showed moderate to strong correlation between the assessments for animal EF (**A**) and GLS (**B**) with a moderate bias, as shown in the Bland–Altman plots, mean difference (95% CI for limits of agreement) of 1.56 (−4.96 to 8.09) for animal EF (**C**) and −1.01 (−9.30 to 7.28) for animal GLS (**D**). A moderate to strong correlation was also shown between the assessments of the two observers for human EF (**E**) and GLS (**F**) with a moderate bias, as shown in the Bland–Altman plots, mean difference of −4.50 (−12.27 to 3.27) for human EF (**G**) and −1.08 (−5.79 to 3.64) for human GLS (**H**).

The interobserver variability comparison showed moderate to strong correlation between the assessments of the two observers for animal EF (Figure 5A) and GLS (Figure 5B) with a moderate bias, as shown in the Bland–Altman plots, mean difference (95% CI for limits of agreement) of 1.56 (−4.96 to 8.09) for animal EF (Figure 5C) and −1.01 (−9.30 to 7.28) for animal GLS (Figure 5D). A moderate to strong correlation was also shown between the assessments of the two observers for human EF (Figure 5E) and GLS (Figure 5F) with a moderate bias, as shown in the Bland–Altman plots, mean difference of −4.50 (−12.27 to 3.27) for human EF (Figure 5G) and −1.08 (−5.79 to 3.64) for human GLS (Figure 5H). The repeatability of the interobserver assessments fluctuated between good ICC (95% CI) for interobserver animal EF assessments: 0.89 (0.69 to 0.96) and 0.63 (−0.04 to 0.88) for human EF, 0.68 (0.31 to 0.88) for animal GLS, and 0.73 (0.39 to 0.90) for human GLS interobserver assessments.

## 3. Discussion

The aim of this study was to compare cardiac alterations in a transgenic rat model for PE with the human post-PE situation. In order to describe cardiovascular changes after pregnancy in both species, STE was performed by blinded observers. The reported data reflect equivalent changes in the transgenic rat model and the human situation. Functional parameters such as EF and GLS were reduced in both species post-PE. We observed an increase in relative wall thickness and an increase in the posterior wall thickness of the LV as signs of structural remodeling. Melchiorre et al. were among the first who described permanent cardiovascular changes concerning relaxation abnormalities, diastolic dysfunction, and alterations according to LV geometry during and after PE in humans [4,18,19]. They evaluated LV dysfunction and geometry according to the European Association and American Society of Echocardiography guidelines, not mentioning the individual parameters that were altered. The novelty of our study is based on the very sensitive method of measuring myocardial function using STE. Former preeclamptic women often suffer from asymptomatic cardiac abnormalities with prevalence of asymptomatic heart failure stage B including concentric remodeling and mildly impaired EF [6]. STE offers more sensitive parameters by describing the deformation of the myocardium and is even able to distinguish between individually contracting muscle layers of the heart. Previous publications demonstrated the extensive ability of STE to differentiate between physiological and pathological hypertrophic changes of the heart [20]. With the unique possibility of targeted myocardial specification, STE is being used more frequently in animal models [21,22,23]. The GLS, as the most sensitive parameter, was induced much earlier than EF [24] and showed a reduction of cardiac functionality post-PE in both species of our translational comparison. To our knowledge, this is the only animal model investigating cardiovascular changes postpartum by STE. An important open and unacknowledged question is whether the changes seen in humans are reversible [25] or permanent after delivery [26], and, if this is going to be an irreversible remodeling, whether replacement fibrosis is also present. We observed perivascular and interstitial fibrosis in the transgenic animal model [16], suggesting that a persisting structural remodeling post-PE and thus permanent cardiovascular abnormalities after preeclamptic pregnancy might be present after human preeclamptic pregnancy. Cardiovascular magnetic resonance (CMR) displays a high potential in the detection of myocardial fibrosis. One of our latest publications about CMR in formerly preeclamptic women describes the structural remodeling postpartum and underlines the burden of increased cardiovascular risk in later life. In this four year postpartum study, diffuse injury of the myocardium was assessed by parametric mapping, focal injury by late gadolinium enhancement imaging, and cardiac function was evaluated by cine imaging and tissue tracking (strain). The post-preeclamptic group showed increased left-atrial end-diastolic volume stroke volume with a slight increase in left ventricular hypertrophy. We could not detect differences in focal or diffuse myocardial tissue composition between the groups. Follow up studies are needed to identify the critical window when morphologic tissue differentiation, such as progression to myocardial fibrosis or inflammation, occurs in post-preeclamptic patients. Beside replacement fibrosis, another indication for permanent alterations in the maternal heart is the concentric remodeling. LV posterior wall and the extrapolated relative wall thickness increased in this study in both post-PE species. Intriguingly, the LV mass only increased significantly in the animal model. However, already published human studies show a permanent hypertrophy of the maternal heart [4,18,27]. 

The importance of PE as a risk factor in the development of chronic heart disease has been reinforced, and the future cardiovascular health of formerly preeclamptic women is getting more and more attention. Since 2011, the American Heart Association concludes that a pathological pregnancy is an important novel risk factor for later CVD [1]. While California was the only state in the USA to reduce maternal mortality due to hemorrhage using disease-specific toolkits and evidence-based protocol [28], death rates from maternal CVD are rising in the rest of the country. That demonstrates the urgent need for a cardiovascular follow-up after preeclamptic pregnancy and a preventive strategy worldwide. New guidelines from the European Society of Cardiology for the management of CVD during pregnancy [29] recommend lifestyle modifications and annual visits to a primary care physician to check blood pressure and metabolic factors. A sufficient strategy for cardiovascular prevention and postpartum treatment is still missing. To investigate the disease related remodeling process of the maternal heart, animal models are essential. The transgenic rat model mimics a preeclamptic pregnancy in several important aspects [11,12,13,14,15] and shows characteristic features of cardiac dysfunction after a preeclamptic pregnancy, as shown in this study.

In conclusion, our data indicate that the transgenic rat model and the use of STE depicts the cardiovascular abnormalities in the human situation well. That provides the basis investigating the causes of structural changes and functional disorders and gives the opportunity to test promising interventions. To underpin the methodical safety, we used one single observer for STE analysis in both species and demonstrated excellent intraobserver comparison. Interobserver agreements differed between EF and GLS and underlined the important aspect of fixed observers within a study. However, it should be mentioned that preeclampsia is not a monocausal disease, and there is no model that completely reflects the heterogeneous clinical picture of this disorder. Another limitation is the postpartum observation time point of four weeks in the animal model. Further studies with longer postpartum periods corresponding to 10–20 human years will be necessary in the future to be able to make further statements about the cardiovascular risk after a preeclamptic pregnancy. 

## 4. Materials and Methods 

### 4.1. Animal Cohort

A transgenic rat model was used for simulation of a preeclamptic pregnancy. Female Sprague-Dawley rats harboring the human angiotensinogen gene [TGR(hAogen)L1623] were mated with male rats transgenic for the human renin gene [TGR(hRen)L10J] and developed the disease related phenotype during the last trimester of pregnancy. Age- and body weight-matched wild-type Sprague-Dawley rats constituted the control group. Both groups were housed in a temperature-controlled environment of 22 ± 2 °C, 12:12-hour light/dark cycle, and a humidity of 55 ± 15%. The animals had access to water and food (Sniff V1324-300) ad libitum. Rats were sacrificed 28 days postpartum by decapitation with prior isoflurane anesthesia or due to predefined stopping. The study was approved by local authorities (G0273/16; State Office of Health and Social Affairs Berlin).

### 4.2. Animal Echocardiography

Transthoracic echocardiography in both groups (control *n* = 8, PE *n* = 8) was performed postpartum in anesthetized animals (1.5% isoflurane via an oxygen mask). Temperature, ECG, and respiration were monitored. By a heated platform, rectal temperature was maintained at 37 ± 2 °C. Abdominal hair was removed by depilatory cream. Pre-warmed gel was used as an ultrasound-coupling medium. For imaging, a Vevo3100 high-resolution imaging system (Fujifilm, VisualSonics Inc., Toronto, ON, Canada) mounted with a 21 MHz transducer (MS250) was used. All images were acquired and stored for offline analysis. Analysis was done by two blinded observers using VisualSonics VevoStrain software (Version 2.2.0, Toronto, ON, Canada). In parasternal long and short axis view, B-Mode cine loops were used to assess speckle tracking analysis. Images were checked for quality with regard to absence of artifacts and differentiation of wall borders. The endocardium of the left ventricle was traced manually in end-diastole. The epicardium was automatically traced by the software, checked and manually adjusted if necessary, for maximum tracking accuracy. Global strain values were obtained from the average of the six segments of the left ventricle. M-mode was obtained to measure cardiac wall and chamber dimensions. Relative wall thickness was calculated by the formula (2*PWd)/LVEDD. Analysis was performed on three consecutive cardiac cycles; mean values from three measurements were calculated.

### 4.3. Human Cohort

This prospective case-control study was carried out at St. George’s University Hospitals NHS Foundation Trust in London over a 12-month period from April 2016 until March 2017. The local institutional review committee approved the study (ID 12/LO/0810; NRES Committee London—Stanmore; date: 30-06-2012), and all participants provided written informed consent. Women with singleton pregnancy were recruited as cases. There were no cases of primigravida. Only women without any cardiovascular co-morbidity and before starting any antihypertensive medication were asked to take part in the study. Preeclampsia was defined according to the guidelines of the International Society for the Study of Hypertension in Pregnancy (ISSHP) [30,31]. Normotensive healthy term pregnant women without any co-morbidity were recruited as controls. Blood pressure was measured manually from the brachial artery according to the guidelines of the National High Blood Pressure Education Program Working Group on High Blood Pressure in Pregnancy [32]. Maternal data of the human cohort are given in Table 1.

### 4.4. Human Echocardiography

Echocardiographic examination and analysis were performed by a single operator (BSB) using a GE Vivid Q ultrasound machine equipped with a 3.5 MHz transducer (GE Healthcare, Boston, MA, USA). Images were acquired at rest in the left lateral decubitus position from standard parasternal and apical views. Digital loops of 3 cardiac cycles with associated electrocardiogram information were stored on the hard disk of the ultrasound machine and transferred to a GE EchoPac workstation (GE Healthcare, Boston, MA, USA) for offline analysis. Analysis was performed according to existing guidelines [33]. Interventricular septum thickness, left ventricular posterior wall thickness, and left ventricular systolic and diastolic diameter were measured in the parasternal long axis view. Left atrial volume (LAV) and left ventricular volume in diastole (LVEDV) were calculated from apical views. Left ventricular mass was calculated using the Devereux formula 0.8(1.04[([LVEDD + IVSd + PWd]^3^ − LVEDD^3^)]) + 0.6v, where LVEDD is left ventricular end diastolic diameter, IVSd is thickness of the intraventricular septum in diastole, and PWd is posterior wall thickness in diastole. Relative wall thickness was calculated with the formula (2*PWd)/LVEDD. For speckle tracking echocardiography, the myocardium was traced manually, and the EchoPac software then suggested an area of interest by delimiting the endocardium and the epicardium. The operator readjusted this area before the software calculated deformation. LV endocardial and epicardial global strain as well as LV longitudinal, LV early, and LV late diastolic strain rates were calculated from apical views. Negative values indicate fiber shortening, and positive values indicate fiber lengthening. If >1 segment was rejected, subjects were excluded from statistical analysis.

### 4.5. Statistics

Statistical analyses were performed by using SPSS version 25 (IBM, Armonk, NY, USA) and Prism 7.0 software (GraphPad Software Inc., San Diego, CA, USA). ROUT method was performed for outlier identification with an average false discovery rate less than 1%. After testing for normal distribution group differences were analyzed by 2-tailed unpaired t-test or by Mann–Whitney U test. All data are presented as means ± SEM. To describe the quality and the sensitivity of the STE measurements in both species, intra- and interobserver comparisons were presented for EF and GLS. Intra- and interobserver variability was assessed on all animals and all study participants by the repeated evaluation of the EF and GLS. Pearson correlation, Bland–Altman plots, and intraclass correlation were performed to assess the agreement and the reliability within and between the observers. For interobserver comparison, two experts evaluated parameters of EF and GLS independently in a blinded manner concerning the animal model as well as in the human participants. For intraobserver comparison, one observer repeatedly evaluated EF and GLS in a blinded fashion. Bland–Altman plots were reported including the mean difference (bias) with corresponding 95% limits of agreement between the evaluations of the two observers (interobserver agreement) or the repeated evaluations of one observer (intraobserver agreement). For the calculation of the intraclass correlation coefficient, a two-way random-effect model based on single measurements and absolute agreement assessed the interobserver repeatability, and a two-way mixed-effect model based on single measurements assessed the intraobserver repeatability for one observer. Mean estimators with 95% confidence intervals (CI) were reported for each ICC. Interpretation of ICC and Pearson correlation coefficients was elaborated as the following: <0.50 = poor; between 0.50 and 0.75 = moderate; between 0.75 and 0.90 = strong; >0.90 = excellent. A two-sided *p* < 0.05 was considered statistically significant.

## 5. Conclusions

The use of STE has shown that the transgenic animal model reflects the changes after preeclamptic pregnancy in a human situation. By applying the state-of-the-art technique STE, especially marginal changes can be detected due to its sensitivity. This will be of paramount importance for future studies on the cause of early cardiac remodeling and the evaluation of potential interventions.

## Figures and Tables

**Table 1:** Maternal data of human cohort. Cases and controls are matched in age, body mass index (BMI), and scan time after delivery. Preeclamptic women show lower gestational age of delivery. Data given as mean ± SEM.

	Control (*n* = 8)	Preeclamptic (*n* = 8)	*p*-Value
Age (years)	36.3 ± 1.71	35.0 ± 1.45	0.5860
Weight (kg)	70.9 ± 5.2	77.8 ± 7.3	0.4569
Height (m)	1.7 ± 0.0	1.6 ± 0.0	0.2014
BMI (kg/m²)	25.1 ± 1.58	28.8 ± 2.17	0.1870
Gestational age of delivery (weeks)	40.4 ± 0.60	31.5 ± 1.49	<0.0001
Scan after delivery (weeks)	45.5 ± 5.45	49.8 ± 3.34	0.5169

## Abbreviations

CVD	Cardiovascular disease
EF	Ejection fraction
GLS	Global longitudinal strain
hAogen	Human angiotensinogen
hRen	Human renin
ICC	Intraclass correlation coefficient
IVSd	Intraventricular septum diastole
LV	Left ventricle
LVEDD	Left ventricle end diastolic diameter
ns	Non-significant
PE	Preeclampsia
PWd	Posterior wall diastole
SEM	Standard error mean
sFlt-1	Soluble fms-like tyrosine kinase I
STE	Speckle Tracking Echocardiography
